# Impact of ultrasound–enzyme pretreatment sequence on recovery and functionality of proteins from an oat fiber-rich side stream

**DOI:** 10.1016/j.fochx.2025.103452

**Published:** 2025-12-28

**Authors:** José Villacís-Chiriboga, Helga Guðný Elíasdóttir, Isabel Badager, Mehdi Abdollahi

**Affiliations:** Department of Life Sciences, Food and Nutrition Science, Chalmers University of Technology, SE-41296 Gothenburg, Sweden

**Keywords:** Ultrasonication, Bioprocessing, Oat, Plant-based protein, Side streams

## Abstract

The residual fiber-rich fraction after oat protein extraction is a promising resource for sustainable protein recovery, though its rigid protein–fiber matrix hinders extraction. This study assessed effects of mechanical (wet milling, ultrasound) and biochemical (Viscozyme®) pretreatments, individually and in sequence, on its protein recovery using alkaline extraction and isoelectric precipitation. Wet milling slightly improved yields *via* particle size reduction, while ultrasound alone enhanced protein purity. The combination of ultrasound and enzymatic treatment doubled protein recovery and mass yield, also boosting gelation and viscoelastic strength. Pretreatment order was crucial: ultrasound followed by enzyme treatment yielded higher surface charge and gel strength, whereas enzyme-first produced finer protein particles with moderate gel strength. Ultrasound-first treatments showed lowest phytate retention, enzyme-first the highest. These results reveal the synergistic, sequence-dependent effects of ultrasound and enzymatic pretreatments in releasing protein from fiber-rich oat side stream, supporting their upcycling into functional, plant-based food ingredients.

## Highlights

• Ultrasound followed by carbohydrase pretreatment doubled protein yield from oat press cake
• Sequence of treatments critically affected protein structure and gel properties
• Ultrasound enhanced purity, unfolding proteins and reducing phytate retention
• Enzyme-first led to finer particles but increased phytate–protein complexation
• Combined methods offer sustainable route to functional oat protein ingredients

## 1 Introduction

A transition toward plant-based food consumption and production is urgently needed to reduce the environmental burden of the food system while meeting the growing global protein demand (World Food Programme, 2024). The plant-based food sector offers significant opportunities to improve resource efficiency across its production value chains, where all streams are considered valuable and are utilized as food, feed, or bioethanol—ensuring that no material goes to waste. Plant-based side streams are, however, complicated by factors such as imbalanced nutrient composition, complex structural properties, and the presence of anti-nutrients and off-flavor compounds (Sha & Xiong, 2020).

Among plant-based food resources, oats (*Avena sativa* L.) stand out due to their adaptability to cold climates, with global production and consumption exceeding 23 million metric tons in the 2024/2025 period (USDA, 2025). The nutritional composition of oats includes starch (60%), protein (11–15%), lipids (5–9%), dietary fiber (2.3–8.5%), and smaller amounts of vitamins and minerals (Rasane, Jha, Sabikhi, Kumar, & Unnikrishnan, 2015). Several studies have associated the positive nutritional impact of regular oat consumption (*i.e.*, triglycerides and cholesterol reduction, and improvement in cognitive function) with its soluble dietary fiber content, particularly β-glucan (Amerizadeh, Ghaheh, Vaseghi, Farajzadegan, & Asgary, 2023; Jibril, Arero, Kankam, & Fuseini, 2023). More recently, the protein fraction of oats has also attracted growing scientific interest (Li & Xiong, 2021). In light of these benefits, impetus have been invested to extract and investigate the dietary fiber-rich and protein-rich fractions from oats. Nevertheless, the extraction of such fractions results in a substantial amount of biomass.

In this context, the insoluble fiber fraction remaining after oat protein extraction, often referred to as “*oat fiber rich fraction*”, has emerged as a promising secondary protein source. While traditionally overlooked, this fraction still contains significant residual protein alongside dietary fiber and bioactive compounds. Its nutritional composition has been reported to include protein (25–30% DW) and total dietary fiber (37–41% DW) as major components, with smaller amounts of starch (13–17% DW) and fat (14–15% DW) (Lindeberg-Lindvet, 2022). Therefore, its recovery and characterization as a nutrient-rich ingredient can support the development of novel food products or supplements, contributing to improved protein utilization and overall process sustainability in oat-based biorefineries. Nevertheless, extracting protein from such a carbohydrate-rich matrix presents a challenge due to strong protein-polysaccharide interactions, which form complex structures that hinder protein solubility and separation. Additionally, presence of phenolic compounds and anti-nutritional factors can interfere with extraction efficiency, reducing protein yield and functionality in food applications (Dobson, Pensini, Dupuis, Yada, & Marangoni, 2023). Therefore, conventional protein extraction techniques such as alkaline solubilization and isoelectric precipitation must be intensified and tailored using assistance technologies to ensure a high yield, without compromising its nutritional quality or functional properties.

Pretreatment with enzymes could selectively break down the rigid carbohydrate structure-composed of cellulose, hemicellulose, and β-glucans and, thereby facilitate protein release from the (oat) fiber-rich fraction. Enzymes such as cellulases, xylanases, and proteases target specific linkages within the plant cell wall or protein-carbohydrate complexes, improving accessibility and extraction yields (Gupta & Lee, 2013). However, their high cost and specific operating conditions, *e.g.* narrow pH and temperature optima, and the need for prolonged incubation times, complicate their large-scale industrial applications (Gouseti et al., 2023). To overcome these challenges, ultrasound (US) can be an alternative or assistance technology to weaken carbohydrates-protein interactions that obstruct protein release, thereby improving extraction efficiency (Fan et al., 2023). Ultrasound induces cavitation, which occurs when high-frequency sound waves generate microscopic bubbles in a liquid medium. These bubbles rapidly expand and collapse, creating intense localized pressure and temperature fluctuations. Such micro explosions disrupt the cellular structures, weaken protein-polysaccharide interactions, and enhance mass transfer, improving extraction efficiency (Ampofo & Ngadi, 2022). Beyond extraction, the high energy and shear forces induced by cavitation can cause partial protein denaturation by unfolding its secondary structure. This unfolding exposes hydrophobic and hydrophilic functional groups, improving solubility, emulsification, and gelation properties, key functional attributes in food applications (Dash, Singh, & Singha, 2025).

Despite growing interest in enzyme- and ultrasound-based methods individually (Turker & Isleroglu, 2024), limited research has systematically explored their combinations or the sequence in which they are applied to extract protein from the residual fiber-rich fraction after oat protein extraction. However, studies have evaluated their synergistic effects in other plant matrices with opposite behaviors. On one side, Zhao et al. (2023) examined the effect of multi-frequency ultrasound-assisted cellulase treatment on the extraction and quality of mulberry leaf protein. The authors concluded that ultrasound treatment exposed hydrophobic groups and induced molecular unfolding, thereby enhancing the binding affinity of cellulase to substrates, reducing its interference effect and consequently promoting protein solubilization. In a different perspective, Turker and Isleroglu (2024) reported that enzymatic pretreatment with a multi-enzyme complex rich in carbohydrases, followed by ultrasound treatment, significantly increased cress seed protein yield from 39.56% to 94.03%, demonstrating the effectiveness of this combined approach. Thus, a clear gap remains in understanding the mechanistic interactions and optimal sequencing strategies of ultrasound and enzymatic treatments for efficient and sustainable protein recovery from oat-derived side streams. It is hypothesized that applying ultrasound before enzymatic treatment may increase substrate accessibility by disrupting cell walls, whereas post-enzyme ultrasound may enhance diffusion and facilitate product release. Conversely, synergistic effects may also depend on enzyme sensitivity to shear forces and local heating.

With these considerations, this study aimed to evaluate the effect of US in combination with Viscozyme®, a cellulolytic enzymatic cocktail, and their sequence on protein extraction efficiency from the oat fiber-rich fraction using alkaline solubilization and isoelectric precipitation and its impact on protein quality. The key questions were (i) whether the synergistic action of US and enzymatic treatment in disrupting and weakening the fibrous structure would enhance protein recovery during wet fractionation, (ii) how the sequence of applying US and enzymatic treatment affect protein recovery and (iii) how ultrasound/enzyme-assisted extraction affect the quality of the recovered proteins in terms of particle size, zeta potential, polypeptide pattern, water solubility, emulsification capacity, and rheological properties. Additionally, the potential influence of the treatments on the phytate content of the extracted proteins was investigated.

## 2 Materials and methods

### 2.1 Raw materials

Oat processing side stream called insoluble oat fiber fraction from production of protein-rich and fiber-rich ingredients were provided by Lantmännen (Sweden) in the wet form with 78% moisture. The sample was then frozen and stored at −80 °C until further processing. The multi-enzyme blend of Viscozyme® was purchased from Novoenisis.

### 2.2 Wet milling with high-shear mechanical homogenization

The oat fiber fraction was mixed with distilled water (1:7 *v*.*w*^−1^) and milled with a high-shear mechanical homogenizer (HSMH) (LM5, Silverson, Massachusetts, US) at 10800 rpm, equipped with a radial discharge head. The process was tested for 3 and 10 min to get different milling levels and particle sizes. To prevent excessive heating during milling, the samples were put in close contact with cold water and ice cubes.

For small scale processing, 30 g were suspended in 210 mL distilled water (1:7 *w*.*v*^−1^). The resulting material was used to determine protein purity, mass and protein yield. For large-scale processing (used in chemical and functionality tests), 100 g of oat fiber was combined with 700 mL of distilled water per batch, and milling was performed in seven replicates, resulting in a total of 700 g of oat fiber dispersed in 4900 mL of water.

As conventional extraction (solubilization at pH 11.0 and precipitation at pH 4.5) resulted in negligible protein recovery due to the coarse rigidity of the fiber matrix, HSMH was evaluated as a pretreatment to disrupt the structure and improve protein accessibility. The 3-min milling condition, which showed a positive outcome, was established as the control, representing the baseline level of mechanical processing prior to protein extraction and precipitation (Section 2.4). This duration was selected considering the potential scalability of the process and the challenges associated with maintaining low temperatures during extended homogenization, as prolonged milling (*e.g.*, 10 min) could lead to heat buildup and protein denaturation, making industrial implementation more difficult.

### 2.3 Pretreatment with ultrasound, enzyme and their combinations

#### 2.3.1 Ultrasound-assisted extraction

The 3-min HSMH sample was subjected to ultrasound treatment for 25 min (in cycles of 10 s on/40 s off), at 75% amplitude while kept on an ice bath, using a 20 kHz probe ultrasonicator (UIP 1000hdT, Hielscher, Ultrasound Technology, Germany) with a max power of 145 W and equipped with a 10 mm diameter sonotrode. The sample was incubated for 30 min after the US treatment, keeping a stable pH of 11.0 using 1.0 N NaOH while stirring. The mixture was then subjected to protein extraction and precipitation as explained in Section 2.4. For the small scale, the entire homogenate was used while for the large scale, the mixture was divided into two equal fractions of 2.8 kg each.

#### 2.3.2 Enzyme-assisted extraction

The oat fiber fraction was dispersed in distilled water at a 1:7 *w*.*v*^−1^ and milled for 3 min using the HSMH. The pH of the resulting homogenate was adjusted to 4.6 with 1.0 N HCl and stirred for 10 min using a magnetic stirrer in the small-scale experiment, while in the large-scale process, an overhead mixer was employed. Subsequently, the mixture was placed in a 44 °C water bath. Once temperature equilibrium was reached, Viscozyme® was added at a concentration of 30 fungal beta-glucanase units (FBG) per 10 g of oat fiber, and the mixture was incubated for 1.5 h at 44 °C in a water bath with overhead mixing. The pretreated mixture was then used for protein extraction as described in Section 2.4.

#### 2.3.3 Ultrasound-then-enzyme assisted extraction

The sample was pretreated following the same procedure described in Section 2.3.1 for ultrasound-assisted extraction. Then, the enzymatic hydrolysis was carried out following the procedure explained in Section 2.3.2.

#### 2.3.4 Enzyme-then-ultrasound assisted extraction

The enzymatic hydrolysis of the fibrous cell wall was achieved following the procedure described in Section 2.3.2. The resulting material was subjected to US-assisted extraction, as explained in Section 2.3.1.

### 2.4 Protein extraction and precipitation

To separate the solubilized protein in the samples, the suspension was centrifuged (5500×*g* for 20 min at 20 °C) and sieved to separate the pellet from the supernatant. Then, the pH of the supernatant was lowered to 4.5 with HCl 1.0 N, and stirred for 10 min. The pellet containing oat protein was then collected by centrifugation (5500×*g* for 20 min at 20 °C), weighed and freeze-dried.

### 2.5 Measurement of protein recovery, purity and mass yield

The total nitrogen content was determined using the Dumas combustion method (elemental analyzer, vario MICRO Cube) (Elementar Analysensysteme GmbH, Hanau, Germany) using 2 mg of each recovered protein and the starting material. The total nitrogenous content was then converted to protein using 5.4 as a conversion factor (Mariotti, Tomé, & Mirand, 2019). All analysis was performed in triplicates and expressed in dry matter weight (DW). Protein purity, mass yield and protein recovery were calculated with the following formulas:

(1) $$\mathrm{Protein}\mathrm{purity}=\text{Nitrogen content}\;\left[\%\right]\times 5.4$$

(2) $$\mathrm{Mass}\mathrm{yield}\;\left[\%\mathrm{DW}\right]=\frac{\text{Weight of recovered protein pellet}}{\text{Weight of starting biomass}}\times 100$$

(3) $$\mathrm{Protein}\mathrm{recovery}\;\left[\%\mathrm{DW}\right]=\frac{\text{Weight of recovered protein pellet}\;\left[g\right]\times \text{Protein content of the protein pellet}\;\left[\%\right]}{\text{Starting biomass}\;\left[g\right]\times \text{Protein content of starting biomass}\;\left[\%\right]}\times 100$$

### 2.6 Characterization of recovered oat proteins

#### 2.6.1 Zeta-potential and particle size distribution

Zeta potential (ζ) and particle size readings were conducted with a dynamic light scattering analytical instrument (DLS Zetasizer Ultra, Malvern Panalytical Limited, Worcestershire, UK) according to Li et al. (2023). First, 25 mg of each protein rich powder was dispersed in 25 mL of distilled water (1.0 mg.mL^−1^) (Luna-Valdez et al., 2017). The pH of the samples was adjusted to a desired range (3.0–11.0) using 1.0 M NaOH or 1.0 M HCl and stirred for 30 min. The samples were then diluted to a concentration of 0.1 mg.mL^−1^ using distilled water with the same pH and centrifuged at 5500×*g* for 30 min at 4 °C. The supernatant was used for zeta potential measurement. To measure particle size, samples adjusted to pH 7.0 were used. At neutral pH, protein particles tend to be more stable and less prone to aggregation, which allows for a more consistent hydrodynamic diameter. This stability is essential for obtaining accurate particle size measurements, as aggregation can distort the results (Víctor, Ruiz-Henestrosa, Martinez, Patino, & Pilosof, 2012). The DLS Zetasizer Ultra, could measure particle sizes between 0.3 nm to 10 mm. Zeta potential and particle size measurements were performed in triplicates.

#### 2.6.2 Water solubility

Initially, 500 mg of each oat protein extract was dispersed in 20 mL of distilled water and its pH was adjusted to 3.0–11.0 using 1.0 M NaOH or HCl. The samples were stirred for 30 min at a temperature of 20 °C constantly monitoring the pH to keep it stable. Then, the solutions were centrifuged at 15000×*g* for 30 min at 4 °C and the soluble protein content in the supernatants was determined using the modified Lowry protein determination method using bovine serum albumin as standard (Markwell, Haas, Bieber, & Tolbert, 1978). The protein solubility of the samples was calculated using the following equation:

(4) $$\mathrm{Protein}\mathrm{solubility}=\frac{\text{Protein concentration in supernatant}\;\left(\frac{\mathrm{mg}}{\mathrm{mL}}\right)}{\text{Protein concentration in}\;\mathrm{pH}\;\text{with maximum solubility}\;\left(\frac{\mathrm{mg}}{\mathrm{mL}}\right)\;}\times 100$$

#### 2.6.3 Emulsification properties

Emulsifying activity index (EAI) and emulsion stability index (ESI) of the protein rich powders were analyzed by creating an oil-in-water emulsion. First, 150 mg of each protein was dispersed in 15 mL of distilled water, then the pH of the system was adjusted to 7.0 with 0.1 M NaOH. After this, 5 g of sunflower oil was added and homogenized using an Ultra Turrax homogenizer at 12000 rpm for 3 min in an ice bath, to avoid overheating the sample. EAI was determined by transferring 50 μL of the emulsion from the bottom of the container to 5 mL of 0.1% SDS solution and reading its absorbance at 500 nm using a UV–visible spectrophotometer at room temperature. ESI was evaluated by measuring the absorbance of the emulsion at 500 nm after 10 min. Measurements were performed in triplicates and the EAI and ESI values were calculated based on the following equation:

(5) $$\mathrm{EAI}\;\left(\frac{m^{2}}{g}\right)=\frac{2\times 2.303\times A_{0}\times \mathrm{DF}}{C\times \varphi \times \theta \times 10000}$$

(6) $$E\mathrm{SI}\;\left(\min\right)=\frac{A_{0}}{A_{0}-A_{10}}∆T\;$$

A_0_ is the absorbance at *t* = 0 min, A_10_ is the absorbance at *t* = 10 min, *DF* is the dilution factor, *C* is the initial protein concentration (g.mL^−1^), φ is the volume fraction of oil in the emulsion, θ is the path length of cuvette (1 cm), ∆t is the elapsed time (10 min), ∆A is the absorbance difference between t = 0 and t = 10 min.

#### 2.6.4 Rheological properties

To understand heat induced gelation behavior of the protein samples, an *in-situ* gelation was carried out as described by Sajib, Forghani, Kumar Vate, and Abdollahi (2023). For the assay, 3 g of each powder was diluted in 12 mL of distilled water, the pH was adjusted to 7.0 with 1.0 M NaOH and stirred for 30 min while keeping the pH stable. A sample fraction (∼1–2 g) was loaded on a dynamic rheometer (Paar Physica Rheometer MCR 300, Anton Paar GmbH, Austria) equipped with a parallel-plate geometry with a plate diameter of 25 mm and a plate gap of 1 mm, operated in an oscillating mode. Mineral oil was added to the edges of the sample, and a cover was put on to prevent evaporation. *In-situ* gelation was performed in four steps: ramping up the temperature from 20 °C to 90 °C at a constant heating rate of 5 °C.min^−1^, followed by a constant temperature at 90 °C for 30 min, and then the temperature was ramped down to 20 °C at a rate of 5 °C min^−1^ and finally 10 min of conditioning at 20 °C. The gelation test was done in a linear viscoelasticity region (*i.e.*, 1% strain and 0.1 Hz frequency) of the samples.

An amplitude sweep test was performed consequently after the *in-situ* gelation to determine the strength of the gel formed from each protein sample. The test was performed over a strain range of 0.01–1000% (ramp logarithmic mode) at a constant frequency of 0.1 Hz and at 20°. The yield stress was determined by the crossover point of G' and G" for each sample. Measurements for each protein sample were performed in duplicates.

#### 2.6.5 Surface color

The surface color of the protein samples was examined using a colorimeter (CR-400, Konica Minolta Sensing, Japan). Each sample was poured into a small petri dish (5–6 cm diameter) and subjected to measurement with five replicates (*n* = 5). Measurements were performed in the CIELAB color space (L*, a*, b*) using the following settings: illuminant D65, 2° standard observer, and measurement geometry d/0°. The instrument operates in Specular Component Included (SCI) mode. Calibration was performed using a standard white calibration tile provided by the manufacturer. To maintain a consistent background, samples were placed on a white plastic board (30 cm × 30 cm × 1.3 cm) before each measurement.

#### 2.6.6 Phytate analysis

Phytate was analyzed as inositol hexaphoshate (IP_6_) by high-performance liquid chromatography (HPLC) according to the method of Carlsson, Bergman, Skoglund, Hasselblad, and Sandberg (2001). Inositol hexaphoshate was analyzed because it is the fully phosphorylated form of phytic acid and the predominant inositol phosphate present in plant-derived materials, typically accounting for 60–90% of total inositol phosphates in cereals and legumes. It represents the main storage form of phosphorus and minerals and is the most responsible for phytate's mineral-binding and nutritional effects. Lower inositol phosphates (IP₅, IP₄, *etc.*) mainly arise from degradation or enzymatic hydrolysis during processing (Carlsson et al., 2001; Żyła & Duda, 2025). For this purpose, 0.5 g of each oat protein sample were mixed with 10 mL of 0.5 mol.L^−1^ HCl for 3 h using a laboratory shaker (Heidolph Reax 2; Heidolph Instruments GmbH Schwabach, Germany). Then, 1 mL of each sample was transferred into Eppendorf tubes and centrifuged for 5 min at 12000×*g*, 20 °C, and then the supernatants were transferred to 0.3 mL PP snap ring micro-vials. A rapid analysis of IP_6_ (isocratic eluent) was performed, at a flow rate of 0.8 mL.min^−1^, with 80% of 1 M HCl and 20% milliQ H_2_O. The injection volume was 50 μL, and the analysis time was 7 min for each sample. The analytical system consisted of a PA-100 (4 × 50 mm i.d., particle size 8.5 μm) guard column (Thermo Scientific Dionex, Sunnyvale, CA, USA) and an HPIC CarboPac PA-100 (4 × 250 mm i.d., particle size 8.5 μm) analytical column (Thermo Scientific Dionex, Sunnyvale, CA, USA). The inositol phosphates were detected after post column reaction using UV detection (UV-4075, Jasco, Japan) at 290 nm.

The eluents were mixed with 0.1% Fe(NO_3_)_3_*9H_2_O in a 2% HClO_4_ solution in a post column reactor. The combined flow rate was 1.2 mL.min^−1^. A mixing tee and a homemade reaction coil of crocheted Teflon tube (i.d 0.2 mm, 4.5 m) that had been optimized with respect to reaction time and to avoid peak broadening, was used to get enough reaction time and blending rate. Calculations of peak areas and elution times were done with the software ChromNav. The phytate concentration was calculated based on an external standard with a concentration range of 0.1–0.6 mmol.mL^−1^ and expressed as mg of phytate per g of protein DW.

#### 2.6.7 Sodium dodecyl sulfate-polyacrylamide gel electrophoresis (SDS-PAGE)

The polypeptide profiles of the oat protein samples were determined with SDS-PAGE according to the method by Laemmli (1970). First, 1 g of each sample was mixed with 9 mL of 5% SDS solution and homogenized using Ian KA polytron Ultra-Turrax (T18 basic ULTRA-TURRAX®, IKA, Germany) at 11000 rpm for 2 min. The homogenate was heated in a water bath at 85 °C for 1 h to dissolve the proteins followed by centrifugation (5000 ×*g* for 5 min). The protein content of the supernatant was determined using a modified version of the Lowry protein determination method (Markwell et al., 1978). The samples were then diluted using 5% SDS to reach 4 μg protein per μL, mixed with an equal amount of Laemmli buffer (Bio-Rad, USA) containing 5% β-mercaptoethanol and heated at 95 °C for 5 min using a heater block. After cooling, the samples were centrifuged at 5000 ×*g* for 5 min. Afterward, 20 μL of each sample was loaded onto the gel (4–20% Mini-PROTEAN® TGX™ Precast Protein Gels, BioRad) together with 5 μL of a marker representing a broad range (10–250 kDa) of polypeptide bands. The gel was stained using a 0.02% (*w*.*v*^−1^) Coomassie Brillian Blue R-250 in 50% (*v*.*v*^−1^) methanol and 7.5% (*v*.*v*^−1^) acetic acid for 30–60 min. Destaining was performed using 50% methanol (*v*.*v*^−1^) and 7.5% (*v*.*v*^−1^) acetic acid for 30 min. Quantification of bands was conducted using Bio-Rad Image Lab 6.1.0. software.

### 2.7 Statistical analysis

Results were reported as mean ± standard deviation (SD). Statistical analysis was performed using OriginPro 2023 Academic (64-bit) software (version 10.0.0.154; OriginLab Corporation, Northampton, MA, USA). One-way analysis of variance (ANOVA) was used to determine significant differences between sample groups (*p* < 0.05). Post-hoc comparisons were conducted using the Tukey's comparison procedure to verify significant differences between mean values of the analyzed variables.

## 3 Results and discussion

### 3.1 Effect of extraction method on protein yield and purity

As can be seen in Table 1, increasing the wet milling time using HSMH from 3 to 10 min significantly increased the protein recovery and mass yield by 50%. This could be due to the more effective size reduction of the oat fiber fraction and thereby facilitating the protein solubilization during the first step of the protein extraction process using alkalization. Similar results have been previously reported on the effect of optimum milling and size reduction on protein extraction efficiency from wheat bran (Eliasdottir et al., 2025) and soy okara (Vishwanathan, Singh, & Subramanian, 2011).

**Table 1:** Effect of extraction conditions on protein recovery, mass yield and purity in% wt.

	Protein recovery [%]	Protein purity [%]	Mass yield [%]
3 min HSMH (Control)	8.37 ± 1.07^c^	38.83 ± 3.61^c^	5.84 ± 0.32^c^
10 min HSMH	12.88 ± 0.17^b^	39.32 ± 2.78^bc^	8.88 ± 0.61^abc^
US	13.99 ± 0.29^b^	46.49 ± 0.96^a^	9.70 ± 1.81^ab^
Enzyme	8.61 ± 0.55^c^	40.75 ± 2.56^abc^	6.97 ± 1.46^bc^
Enzyme + US	18.52 ± 2.60^a^	41.09 ± 2.54^abc^	12.11 ± 1.11^a^
US + Enzyme	17.76 ± 0.65^a^	43.89 ± 1.60^ab^	10.80 ± 0.14^a^

HSMH: high-shear mechanical homogenization. US: ultrasound. All the values are expressed as mean (*n* = 3) ± SD. Different small letters in the same column indicate statistically significant differences (*n* = 3; *p* < 0.05; Tukey test).

Applying US alone as pretreatment after milling did not significantly improve protein recovery or mass yield. This could mean that the cavitation effect induced by US was not sufficient to further destructure the oat fiber-rich fraction or boost protein mass transfer under alkaline conditions. This may be attributed to the tight binding of protein with fiber and other components within the oat matrix, as well as the structural complexity of the oat fiber-rich fraction, which can hinder cavitation energy penetration and reduce access to embedded proteins. Multiple studies indicate that while ultrasound can alter the structural properties of proteins, it does not necessarily enhance protein recovery. For instance, ultrasound pretreatment improved protein solubility and foaming properties in oat beverages but did not increase protein recovery (Kwok, Yang, Taheri, & Chen, 2024). However, a study on pea protein extraction has shown that ultrasound can increase protein extraction efficiency when applied at amplitudes between 25% and 35%. Exceeding these values resulted in decreased extraction (Wang, Zhang, Xu, & Ma, 2020). This can potentially explain the poor performance of US when used alone in our study, as it was applied at an amplitude of 75%. Moreover, biomass complexity plays a fundamental role. In the case of fiber-rich oats, the material contains up to 41% fiber (Lindeberg-Lindvet, 2022), while peas contain about 15% (Foley et al., 2025). These differences in fiber content explain the poor performance of US observed in the results of our study.

Enzymatic pretreatment following HSMH processing significantly reduced both protein recovery and mass yield. This reduction may be attributed to the interference caused by carbohydrate fragments released during non-selective degradation by the enzymatic cocktail. These fragments could have impeded protein solubilization or precipitation by trapping or co-precipitating the proteins. Additionally, the enzymatic treatment conditions, particularly heat exposure under acidic pH, may have negatively affected protein solubility.

The combination of enzymatic treatment and US prior to protein extraction resulted in a twofold increase in protein recovery and mass yield, rising from approximately 8.5% and 5.8% in the control to 18% and 12%, respectively, regardless of the treatment order. However, protein purity did not follow the same trend as recovery or yield across the various treatments. Among all methods tested, US pretreatment significantly improved protein purity, reaching up to 46%. When US was applied before Viscozyme®, the purity was approximately 44%, whereas applying US after Viscozyme® resulted in a purity of about 41%. These findings indicate that inclusion of US in the extraction procedure significantly enhances the protein purity obtained from oat fiber.

This enhanced performance can be explained by the complex composition of the oat fiber-rich fraction, which is rich in β-glucans, cellulose, and hemicelluloses, and exhibits strong protein–polysaccharide interactions. The sequential application of ultrasound and enzymatic treatment leverages their distinct mechanisms, allowing them to synergistically disrupt both the physical and biochemical barriers to protein release (Holopainen-Mantila, Vanhatalo, Lehtinen, & Sozer, 2024). Two potential mechanisms may explain the observed synergy, depending on the sequence of application. In the enzyme followed by ultrasound approach, Viscozyme® first degrades the rigid polysaccharide network, reducing structural integrity and partially solubilizing the fiber. This loosening of the rigid carbohydrate matrix enhances the effectiveness of the subsequent ultrasound treatment, allowing cavitation forces to penetrate deeper into the substrate and detach proteins from the fiber matrix more efficiently (Tang, Huang, & Huang, 2023). Conversely, in the ultrasound followed by enzyme approach, ultrasound physically disrupts cell walls and increases the surface area and porosity of the oat fiber-rich fraction particles, making the fiber matrix more accessible to enzymatic hydrolysis. In both cases, the combination of mechanical and biochemical disruption appears to overcome the structural barriers that limit protein release when each method is applied independently. Similar synergistic effects between ultrasound and enzymatic treatment have been reported in other plant-based systems, such as sesame bran (Görgüç, Bircan, & Yılmaz, 2019), and tea residue (Ayim et al., 2018), highlighting the broad applicability of this approach for valorizing fiber-rich agro-industrial side streams.

In contrast, mechanical homogenization, which relies on physical shear forces to disrupt cells, was less effective at inducing such structural transformations, thereby limiting solubility enhancements (Jian Cedric Sow & Du, 2024). This limited impact of HSMH on protein extraction yield has been demonstrated in other plant-based biomasses too (Carullo, Pataro, Donsì, & Ferrari, 2020). The shearing, stretching, and squeezing interactions generated by high mechanical shearing are subtle and require more intense treatments, such as ultrasound (Carullo, Donsì, Ferrari, & Pataro, 2021; Kong et al., 2019). Ultrasound, through acoustic cavitation, can enhance mass transfer and partially break down cell structures. However, ultrasound alone often does not extract proteins efficiently from complex plant matrices because it mainly disrupts cell structures and enhances mass transfer but does not fully break the strong physicochemical interactions binding proteins to other components (Kariyappa, Sethi, & Arora, 2025). As a result, yields plateau or remain low. Therefore, ultrasound is typically used as a pretreatment in combination with other strategies, such as alkaline conditions or enzymatic hydrolysis, to achieve meaningful improvements in protein yield.

In this study, the combined use of ultrasound and enzyme pretreatment produced higher protein yields compared to mechanical homogenization or the application of ultrasound or enzyme alone.

### 3.2 Effect of the extraction method on the polypeptide pattern of different fractions during extraction

**Fig. 1:**
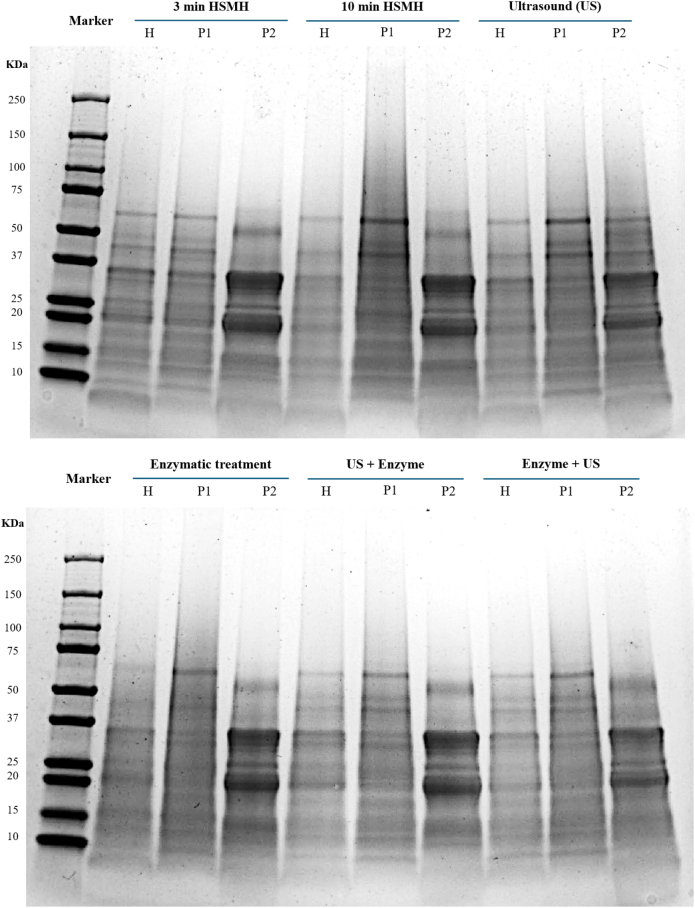
SDS-PAGE patterns of oat fiber-rich residue proteins extracted under the presence of Viscozyme®, US followed by enzyme, and enzyme followed by US, taken at three key stages, alkaline-solubilized homogenates (H), first pellet (P1), and final protein isolates (P2).

SDS-PAGE analysis (Fig. 1) was performed to evaluate the effects of different pretreatments on the polypeptide profile and distribution of oat proteins during the extraction process. Samples collected at three key stages: alkaline-solubilized homogenates (H), the first pellet (P1), and the final protein isolates (P2), revealed generally similar banding patterns across all treatments.

In the P2 fractions, most samples displayed multiple polypeptide bands ranging from 15 to 60 kDa, with two dominant bands observed at approximately 20 kDa and 37 kDa. These are consistent with the molecular weights of major oat globulins and avenins (Mäkinen, Sozer, Ercili-Cura, & Poutanen, 2017). Despite overall similarities, some notable differences emerged. The ultrasound-treated samples exhibited slightly sharper and more intense bands in the mid-molecular weight range (∼30–50 kDa), suggesting improved solubilization and preservation of structurally intact proteins, likely due to cavitation-induced dispersion (Rahman & Lamsal, 2021). In contrast, samples pretreated with enzyme followed by ultrasound (Enzyme + US) showed slightly reduced band intensity in P2. This may indicate that ultrasound applied after enzymatic hydrolysis led to protein aggregation or conformational changes, possibly caused by interactions with degraded carbohydrate fragments, which in turn affected band resolution or protein migration on the gel (Saorin Puton et al., 2025).

This effect was not accompanied by the appearance of lower molecular weight bands or smearing on the gel, suggesting that ultrasound did not induce significant peptide bond cleavage or protein degradation under the conditions applied. This is consistent with literature showing that moderate-intensity ultrasound at 20 kHz typically causes conformational changes and aggregation rather than covalent breakdown in food proteins (Jambrak, Mason, Lelas, Paniwnyk, & Herceg, 2014; Zhang, Regenstein, Zhou, & Yang, 2017).

The remaining treatments, including the control, enzyme-only, and ultrasound followed by enzyme (US + enzyme), exhibited comparable P2 banding patterns, implying that the core protein fractions extracted were similar across these methods, despite the differences observed in extraction yield. Analysis of the H and P1 fractions confirmed that a substantial portion of the protein was solubilized during the alkaline extraction step. However, the P1 fraction still retained a noticeable amount of protein, particularly in the control and enzyme-only treatments, indicating incomplete recovery. Pretreatments involving ultrasound, either alone or in combination, appeared more effective at minimizing protein loss in the P1 fraction.

Overall, while the polypeptide composition of recovered oat proteins remained largely unchanged, ultrasound-based pretreatments enhanced solubilization and dispersion. In contrast, the enzymatic treatment, especially when followed by ultrasound, requires careful optimization to prevent adverse effects on protein structure. This is particularly important when using Viscozyme®, a multi-enzyme cocktail with cellulolytic and hemicellulolytic activities, which may release soluble polysaccharides and phenolic compounds that interfere with protein extraction or lead to co-precipitation (Zhou, Tian, Beltrame, Laaksonen, & Yang, 2023). Viscozyme® effectively hydrolyzes structural polysaccharides in plant cell walls, including cellulose, hemicellulose, and pectin. This enzymatic activity disrupts the complex lignocellulosic matrix, thereby increasing the accessibility of embedded proteins for extraction. For instance, in soy okara, Viscozyme® treatment significantly enhanced protein content and recovery by breaking down cell wall polysaccharides (de Figueiredo, Yamashita, Vanzela, Ida, & Kurozawa, 2018). Similarly, in pea pods, Viscozyme® facilitated protein release by degrading lignocellulosic barriers, resulting in improved protein yields (Karabulut, Yildiz, Karaca, & Yemiş, 2023).

Given the limited improvements in protein yield and purity achieved through HSMH (Table 1), the following sections focus exclusively on enzymatic and ultrasound applications, as well as their combined use, for extracting protein from the oat fiber fraction. However, since the 3-min HSMH treatment was used as the control sample, this sample will be referred to as “Control” in the following sections.

### 3.3 Effect of extraction method on zeta potential, particle size and water solubility of the recovered proteins

**Fig. 2:**
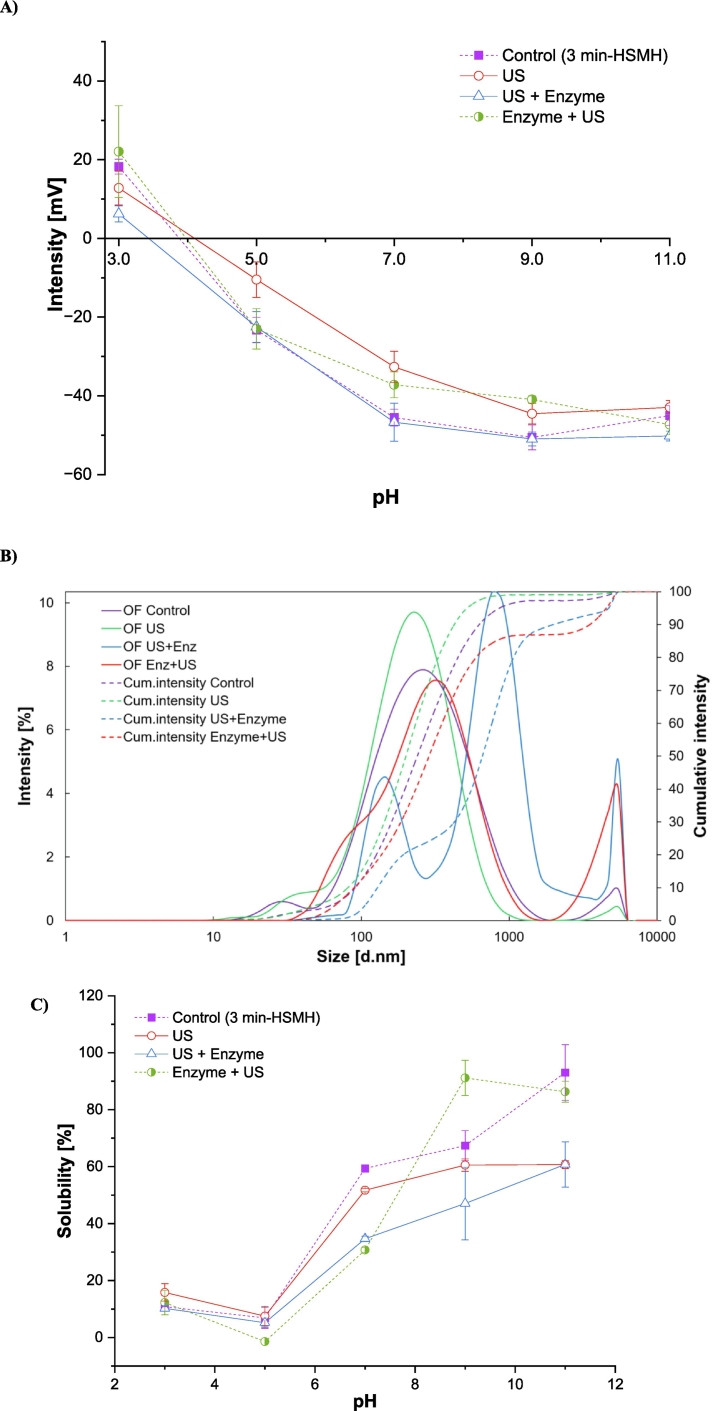
Zeta potential (Fig. 2A), particle size (Fig. 2B) and water solubility (Fig. 2C) of isolated proteins in the presence of Viscozyme®, ultrasound (US) or their combinations (*n* = 3).

The zeta potential and solubility of the isolated oat protein were influenced by pH and by the type of extraction procedure, as depicted in Fig. 2. The zeta potential of all the samples showed a sigmoidal curve, with plateau formation around two pH units outside the isoelectric region, reflecting net positive charge at acidic pH and negative charge at alkaline pH. For most samples, the curve crossed zero between pH 3.9 and 4.1, except for the US + Viscozyme® treatment, which crossed at pH 3.4. These results indicate that the isoelectric point of oat protein lies between pH 3.5 and 4.0 (Fig. 2A). In the control sample, it ranged from 18 to −45 mV, similar to the range observed in the protein extracted with US + enzyme (10.4 to −42 mV). Samples produced with the aid of US and enzyme + US showed sharp differences in their zeta potential at pH 7.0 and 9.0, with significantly lower values compared with the control and US + enzyme, indicating a reduction in net surface charge.

The observed changes in zeta potential can be attributed to the effects of ultrasound on oat protein structure and surface charge. In a protein, the zeta potential range is influenced by factors such as pH, ionic strength, and the intrinsic properties of the proteins, namely amino acid composition, protein structure, isoelectric point (pI), conformation and flexibility, and post-translational modifications (Helmick, Hartanto, Bhunia, Liceaga, & Kokini, 2021). Ultrasound, through cavitation, introduces both mechanical and chemical effects that lead to protein unfolding, aggregation, possibly shielding charged groups or causing conformational rearrangements that decrease surface charge exposure (Wang et al., 2021). These structural modifications significantly affect the zeta potential by altering the distribution and accessibility of ionizable groups.

The sequence of ultrasound and enzymatic treatment significantly affected the surface charge of the extracted oat proteins. The sample treated with ultrasound followed by enzyme (US + enzyme) exhibited higher negative zeta potential values at neutral and alkaline pH (pH 7.0, 9.0, and 11.0) compared to the enzyme + US sequence. This indicates that applying ultrasound first likely disrupted the fiber–protein matrix mechanically, increasing surface area and porosity and thereby improving enzymatic access to protein-bound regions. As a result, the subsequent enzymatic hydrolysis was more effective at liberating proteins and exposing acidic residues (*e.g.*, –COO^−^ groups), which become deprotonated at higher pH and contribute to stronger negative surface charge. In contrast, starting with enzymatic hydrolysis may result in partial degradation of surface polysaccharides, but without prior physical disruption, enzyme action may be restricted to more accessible regions. Ultrasound applied afterward could then cause partial aggregation or rearrangement, limiting the exposure of charged groups. These results highlight the synergistic and sequence-dependent nature of ultrasound–enzyme treatment is more effective for generating highly charged, and potentially more soluble and functionally active oat protein fractions. This results in more substantial modifications to surface charge characteristics, which play a critical role in electrostatic stability and the functional behavior of proteins in solutions.

The particle size distribution of the extracted oat proteins varied significantly depending on the pretreatment strategy applied (Fig. 2b). The control sample exhibited a broad particle size distribution, with a primary peak centered around ∼260–270 nm, reflecting the heterogeneous and aggregated nature of protein–fiber complexes, typical of proteins recovered with alkaline solubilization and isoelectric precipitation. Ultrasound treatment alone resulted in a shift toward smaller particle sizes, with a sharper distribution peak near ∼210–220 nm. This suggests that cavitation and shear forces generated during sonication disrupted some of the larger aggregates and promoted partial disintegration of protein–fiber interactions, in line with previous findings for ultrasound-treated plant proteins (Zhao, Kim, & Eun, 2020). In contrast, the sample treated with ultrasound followed by enzymatic treatment (US + enzyme), displayed a distinct bimodal distribution, with one population below 100 nm and a more prominent peak around ∼1000 nm. Despite partial disintegration, the formation of large aggregates likely reflects protein–fiber re-association or incomplete enzymatic breakdown of larger structures after ultrasound-induced unfolding. This is supported by its cumulative intensity profile, which reached 90% at a much larger particle size (∼2000 nm) compared to other treatments. On the other hand, the enzyme-first followed by ultrasound treatment (Enzyme + US) led to a narrower, more monodisperse particle size distribution centered around 200–300 nm and exhibited the lowest D90 in the cumulative profile, indicating finer dispersion and fewer large aggregates. This outcome can be attributed to Viscozyme® partially degrading the polysaccharide matrix, reducing steric hindrance and improving protein accessibility. The subsequent ultrasound treatment likely enhanced particle disruption and dispersion efficiency, preventing re-aggregation and yielding a more colloidally stable system (Amiri et al., 2021). These particle size results closely align with zeta potential measurements, where the US + Enzyme treatment showed higher surface charge yet more aggregation, and Enzyme + US treatment showed both improved dispersion and higher colloidal uniformity. Together, these findings highlight the strong sequence-dependent synergy between enzymatic and mechanical pretreatments: applying ultrasound after enzymatic loosening of the matrix is more effective at producing homogeneously dispersed and stable protein particles.

**Fig. 3:**
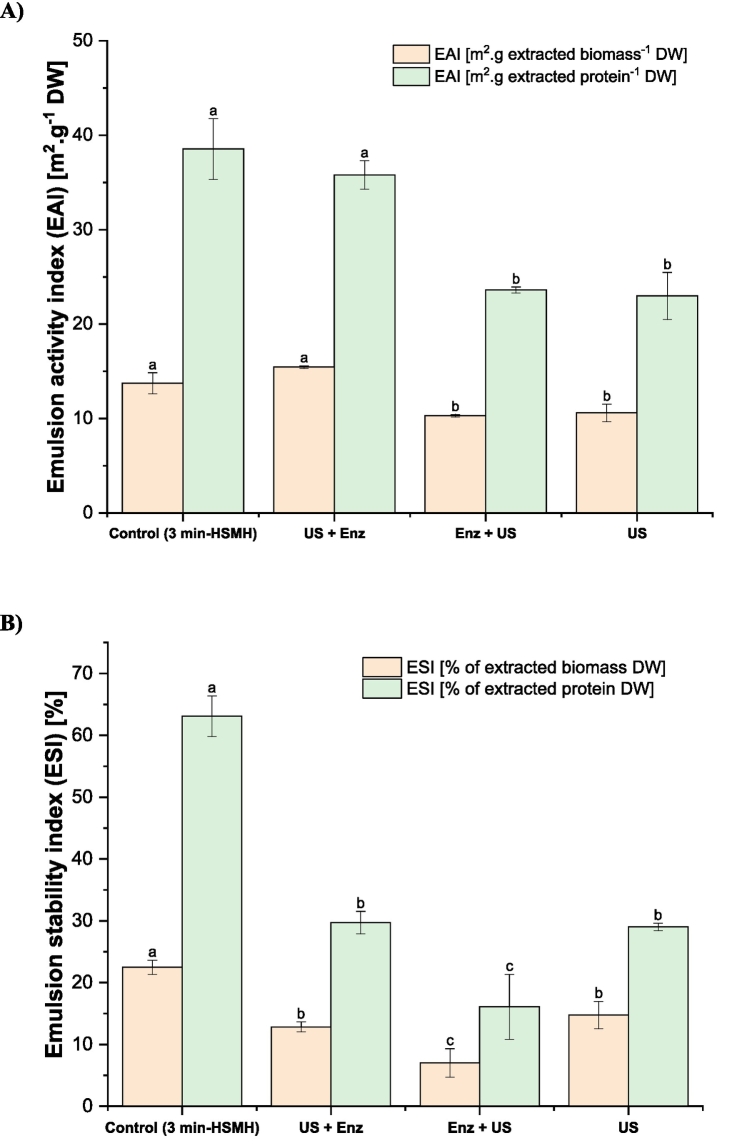
Emulsion activity index (Fig. 3A) and Emulsion stability index (Fig. 3B) of isolated proteins in the presence of Viscozyme®, ultrasound (US) or their combinations. Different small letters indicate statistically significant differences (*n* = 3; *p* < 0.05; Tukey test).

The solubility of oat protein varied significantly with pH and pretreatment strategy as shown in Fig. 3c. All samples exhibited the characteristic U-shaped solubility curve, with the lowest solubility occurring near the isoelectric point (pH 4–5), where electrostatic repulsion is minimal and protein–protein interactions are favored. The control sample demonstrated the highest solubility across the entire pH range, particularly at alkaline pH. Pretreatments involving US, enzyme, or their combinations generally resulted in reduced solubility, especially at neutral and basic pH. Notably, the US-treated sample retained moderate solubility, while the US followed by enzymatic treatment and its counterpart treatments both resulted in markedly lower solubility. These solubility trends were closely linked to changes in zeta potential and particle size. While both the control and US + Enzyme samples displayed relatively high negative surface charges (up to −45 mV) at alkaline pH, the US + Enzyme sample paradoxically exhibited low solubility, indicating that high surface charge alone was insufficient to maintain colloidal stability. This contradiction was clarified by particle size analysis: the US + Enzyme sample showed a bimodal size distribution, including large aggregates around ∼1000 nm, and a broad cumulative intensity profile extending to ∼2000 nm. In contrast, the Enzyme + US treatment produced smaller, more monodisperse particles (∼200–300 nm) and the lowest D90 value, suggesting effective dispersion due to enzymatic loosening of the matrix followed by ultrasound disruption. However, this treatment also resulted in lower zeta potential at neutral and alkaline pH, reducing electrostatic repulsion and limiting solubility. The US-only sample exhibited a sharper, narrower size distribution (∼210–220 nm), indicating some disintegration of aggregates, and retained moderate zeta potential, aligning with its intermediate solubility profile. These findings demonstrate that the solubility of the recovered proteins was governed not solely by surface charge or particle size, but by the interplay between both factors. Ultrasound and enzymatic pretreatments influenced protein conformation, surface accessibility of charged groups, and aggregation behavior in a sequence-dependent manner. While ultrasound enhanced enzymatic accessibility when applied first, it also promoted structural rearrangements that favored aggregation. Conversely, applying ultrasound after enzyme treatment improved dispersion but insufficiently exposed charged residues (Gul, Gul, Baskıncı, Parlak, & Saricaoglu, 2023).

### 3.4 Effect of extraction method on protein functionality

The emulsion activity index (EAI) and emulsion stability index (ESI) results are shown in Fig. 3A and B, respectively. Both EAI and ESI were significantly higher when expressed per gram of protein compared to per gram of biomass (*p* < 0.05). On average, EAI and ESI were 2.35 times greater in the protein fraction than in the biomass.

For EAI in the protein fraction, the control sample and the sample extracted from oat fiber pretreated with US followed by Viscozyme® exhibited the highest values (38.54 and 35.80 m^2^.g protein DW, respectively). In contrast, the samples pretreated with Viscozyme® followed by US and US alone showed lower EAI values, averaging 23.30 m^2^.g protein DW.

The higher EAI in the control sample and in the protein extracted from oat pretreated with US followed by Viscozyme® could be due to the higher concentration of surface-active proteins, which enhances their ability to adsorb at the oil–water interface and form a cohesive interfacial film. Additionally, the US followed by Viscozyme® pretreatment may have promoted partial unfolding of proteins and exposure of hydrophobic regions, improving their emulsifying capacity (Villacís-Chiriboga et al., 2023). In contrast, proteins obtained after Viscozyme® followed by US and US alone exhibited lower EAI values, likely due to structural aggregation or interference from non-protein components. When Viscozyme® was applied first, the enzymatic hydrolysis of cell wall polysaccharides may have increased the presence of soluble carbohydrates and phenolics in the extract, which compete at the interface and reduce protein effectiveness (Liu, Hao, Chen, & Yang, 2020). While US alone may have released proteins with more intact native structures and limited flexibility, restricting their ability to stabilize emulsions compared to partially unfolded proteins. These findings suggest that both the order of pretreatments and the resulting protein purity and structural modifications play a critical role in determining emulsifying properties.

Considering that the method to determine EAI has not been standardized, scientific literature reports different values depending on factors such as oil-to-protein ratio and processing conditions. Some studies estimate EAI by measuring the volume of each phase in the emulsion and monitoring changes over time. However, the method used in this study, originally developed by Pearce and Kinsella (1978) relies on spectrophotometric measurement of diluted emulsions immediately after homogenization, enabling calculation of the interfacial area stabilized by proteins. This approach provides a more quantitative and reproducible assessment compared to volume-based methods, as it accounts for droplet size and surface area rather than relying solely on phase separation. In line with this, a recent study by Peng et al. (2026) evaluated the effect of deep eutectic solvents on various functional properties of oat protein. The EAI and ESI reported by these authors were significantly lower than the values obtained in the present study, despite their higher protein content (80%). Specifically, they reported EAI values below 8 m^2^·g^−1^ protein and emulsion stability not exceeding 36%.

The higher EAI values observed in our samples, despite their lower protein content (≤46%) compared to the 80% reported by Peng et al. (2026), can be attributed to differences in protein functionality rather than total protein concentration. Emulsifying properties depend primarily on the solubility and surface activity of proteins, which govern their ability to adsorb at the oil–water interface. Our extraction method, based on the classical pH-shift process after cell wall disruption, likely produced proteins with greater flexibility and partial unfolding, favoring rapid interfacial adsorption and film formation. In contrast, Peng et al. (2026) reported that their oat proteins maintained a spherical structure after treatment with deep eutectic solvents, which may limit interfacial rearrangement and reduce emulsifying efficiency despite higher purity. Additionally, the presence of non-protein components such as polysaccharides or phenolic compounds in our samples could also contribute synergistically to emulsion stabilization. Finally, differences in pH and ionic strength during emulsification may have enhanced electrostatic repulsion in our system, preventing flocculation and improving dispersion. These factors collectively explain why protein quality and structural adaptability often outweigh protein content in determining emulsifying performance.

Regarding the effect of US over EAI, at 75% amplitude and 20 kHz, US alone effectively disrupted cell walls to release proteins but appeared to preserve their native globular conformation, limiting molecular flexibility and exposure of hydrophobic residues essential for rapid interfacial adsorption and stable emulsion formation. This outcome contrasts with numerous studies reporting EAI enhancements, where US is typically applied to pre-extracted protein isolates or dispersions rather than during extraction from complex matrices (Igartúa, Bernáldez-Sánchez, Palazolo, & Cabezas, 2026; Wang et al., 2020). In those cases, cavitation-induced shear forces and localized turbulence promote controlled partial denaturation, increasing surface hydrophobicity and amphiphilicity without excessive aggregation. In the present oat system, the rigid β-glucan-rich cell walls likely required the applied US energy primarily for mechanical disruption, leaving insufficient intensity or duration for significant tertiary structure modification of the released proteins. However, when US preceded Viscozyme® treatment, initial cavitation may have induced subtle unfolding, facilitating subsequent enzymatic hydrolysis of polysaccharides and yielding purer, more flexible protein fractions with superior emulsifying properties. Conversely, enzymatic pretreatment first released competing soluble carbohydrates and phenolics that interfered at the interface, while US alone co-extracted non-protein components without adequate structural alteration.

Interestingly, the EAI did not directly mirror the ESI, except for the control sample, which exhibited both the highest emulsifying activity and stability (63.09%). For the other treatments, ESI was considerably lower despite relatively high EAI values, with US followed by Viscozyme® and US alone showing intermediate stability (∼29%), and Viscozyme® followed by US presenting the lowest stability (16.08%). This discrepancy suggests that while EAI reflects the initial ability of proteins to adsorb at the oil–water interface, ESI depends on the structural integrity and interactions within the continuous phase over time. Proteins extracted after Viscozyme® followed by US may have undergone extensive hydrolysis and aggregation, reducing their capacity to form a strong interfacial network and resist droplet coalescence (Akbarbaglu et al., 2023). Similarly, US alone may have induced partial denaturation without sufficient unfolding to stabilize emulsions long-term. In contrast, the control proteins likely retained a balanced structure, enabling both efficient adsorption and formation of a cohesive interfacial film, which explains their superior stability. Therefore, pretreatment order influences not only emulsifying capacity but also the resilience of the protein network required for sustained emulsion stability.

Focusing the analysis on the extracted biomass, the ultrasound-only (US) sample exhibited a reduced EAI (10.59%) and ESI (14.78%). This aligns with its solubility profile, which was lower than the control at neutral and alkaline pH. Although ultrasound treatment reduced particle size (∼210–220 nm) and maintained a relatively high surface charge, it likely induced conformational changes, such as protein unfolding, partial denaturation, or aggregation, that limited the flexibility and availability of functional groups at the oil–water interface. These structural alterations may have impaired the protein's ability to stabilize emulsions effectively, despite a favourable zeta potential.

The US + Enzyme treatment led to a small increase in EAI, surpassing both the control and US-treated samples. However, this treatment also resulted in a significant reduction in ESI, likely due to protein aggregation post-hydrolysis. As seen in the particle size distribution, this sample exhibited a bimodal profile with large aggregates (∼1000 nm), despite a high surface charge (zeta potential ∼ −42 mV). The formation of such aggregates limits the ability of proteins to form stable, cohesive interfacial films, leading to instability over time. Previous studies have shown that decreased protein solubility impairs emulsion stability by reducing protein adsorption at the oil-water interface, and larger protein particles, due to slower diffusion and weaker interfacial packing, further compromise stability, particularly in plant protein-based emulsions (McClements, 2009; Zhang et al., 2023). By contrast, the enzymatic treatment followed by US resulted in further reduction of both EAI and ESI and yielded the lowest values among the treatments. Although this treatment produced a narrow and monodisperse particle size distribution (200–300 nm) and the lowest D90, it also showed a marked decrease in surface charge and poor solubility. The ultrasound applied after enzymatic hydrolysis may have further unfolded protein structures, exposing hydrophobic domains and promoting aggregation, while simultaneously reducing the accessibility of ionizable groups critical for maintaining electrostatic stability. These factors combined to severely compromise the proteins' ability to adsorb at oil–water interfaces and maintain stable emulsions.

Overall, these results demonstrate the sequence-dependent synergy and antagonism between ultrasound and enzymatic treatments. While US followed by enzyme appears to improve initial EAI by enhancing enzymatic exposure of interfacial residues, the resulting structural rearrangements and aggregation undermine ESI. On the other hand, enzyme followed by US fails to recover interfacial functionality, likely due to combined effects of over-processing, aggregation, and reduced surface charge. Considering the relatively low purity of the recovered proteins, the role of polysaccharides and other impurities in interpreting the results needs further investigation.

**Fig. 4:**
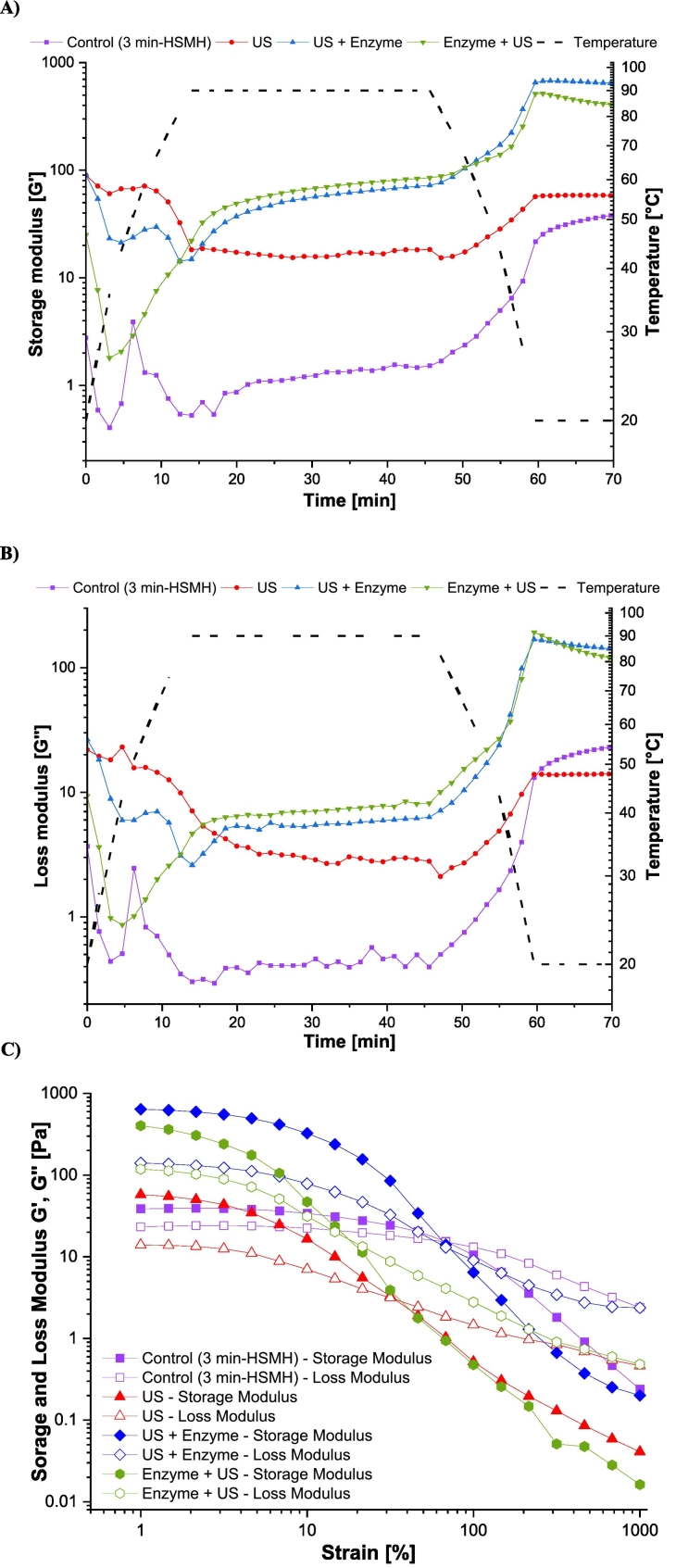
Storage modulus (Fig. 4A), loss modulus (Fig. 4B) and amplitude sweep (Fig. 4C) of isolated proteins in the presence of Viscozyme®, ultrasound (US) or their combinations; (*n* = 2).

The gelation analysis demonstrates that oat protein behaved differently depending on the extraction procedure. The storage modulus (Fig. 4A) [measured in Pa and expressed as G'] reflects the elastic properties of the material, indicating its ability to store energy and resist deformation (Ramli, Asyqin Zainal, Hess, & Chan, 2022). Higher G' values indicate a more solid-gelled structure. G" or loss modulus (Fig. 4B) reflects the viscous properties of the material. The control sample exhibited a minimal increase in G′ and G" throughout the heating and cooling cycle, with values remaining mostly below 10 Pa, indicating poor gelation capacity and a predominantly viscous, weakly structured system (Sun & Arntfield, 2010). This result reflects the limited thermal responsiveness and poor network-forming ability of the untreated oat proteins, likely due to the presence of intact protein–fiber aggregates and a lack of sufficient molecular interactions for stable gel formation. In contrast, all samples treated with a combination of US and enzyme displayed significantly improved gelation behavior, as evidenced by a sharp increase in G′ during heating to 90 °C, followed by further strengthening during cooling. Among them, US followed by enzymatic treatment, produced the highest final G′ values, approaching 700 Pa, suggesting the formation of a strong, elastic protein gel network (Cao et al., 2024). This enhanced gel strength can be attributed to enzymatic degradation of the fiber matrix, which released more proteinaceous material, and subsequent ultrasound treatment, which likely facilitated molecular unfolding and exposure of reactive groups, promoting protein–protein interactions during thermal denaturation (Cao et al., 2024). The enzyme followed by US sample also showed strong gelation, with G′ reaching over ∼510 Pa. In this case, ultrasound likely disrupted the structural matrix initially, improving enzyme access and hydrolysis efficiency, but may have also induced partial aggregation or unfolding that reduced optimal alignment during heating (Sun & Arntfield, 2010). Nonetheless, the high G′ suggests effective crosslinking and network formation. The US-only treated sample exhibited moderate gelation behavior, with G′ values peaking around 50–70 Pa. This implies that ultrasound treatment alone can partially enhance protein functionality by disrupting aggregates and promoting solubilization, but without the additional breakdown of the polysaccharide matrix provided by enzymatic action, its effect on gelation is limited.

The amplitude strain sweep profiles (Fig. 4C) provide insight into the linear viscoelastic region (LVR), indirect insight into gel strength, and structural integrity of oat protein gels as influenced by different pretreatment strategies. The storage modulus (G′) and loss modulus (G″) were monitored as a function of increasing strain, enabling the assessment of gel rigidity and the point at which the network begins to deform irreversibly. The control sample exhibited very low G′ and G″ values across the entire strain range, with G′ and G″ intersecting at a high strain (∼70%), reflecting a weak but highly deformable gel with a wide LVR, consistent with a flexible, loosely structured network that allows significant deformation before yielding, as the protein-fiber aggregates limit rigidity but permit extensibility, aligning the thermal ramp's evidence of poor gelation with the strain sweep's indication of a weak, stretchable gel. The ultrasound-only sample exhibited low viscoelastic properties, with G′ values lower than the control and the combined treatments. Its crossover point (∼30% strain) indicates less gel deformability, reinforcing the notion that ultrasound alone could not improve protein structuring. The enzyme followed by ultrasound treatment showed a strong gel network with high G′ values, although its crossover point occurred slightly earlier than that of the US + enzyme gel (∼15% strain), suggesting a formation of a strong but brittle gel with slightly less structural resistance. The ultrasound-then-enzyme treatment showed the highest initial G′ and G″ values and retained its elastic dominance (G′ > G″) across a broad strain range, up to ∼70–80%, indicating a strong and well-structured gel with a good deformability and viscoelasticity compared with the rest of the sample (Cao et al., 2024). The strong performance of both sequentially treated samples highlights the synergistic role of enzymatic matrix loosening and ultrasound-mediated dispersion and unfolding, which together enhance protein–protein interactions and facilitate network formation.

Overall, the strain sweep results confirm that pretreatment significantly affects the viscoelastic properties and structural robustness of oat protein gels. The US + Enzyme treatment produced the most mechanically resilient gel, followed closely by Enzyme + US, while control and ultrasound-alone treatments showed weaker structures. These differences reflect not only the amount of protein available but also the extent of unfolding, exposure of reactive groups, and the formation of a coherent 3D protein matrix. Such viscoelastic characteristics are critical for food applications where texture, spreadability, and mechanical resistance are key performance attributes.

The functional properties observed in this study highlight the potential of oat protein extracted from fiber-rich fractions as a sustainable ingredient for plant-based food formulations. The high EAI values (up to 38.5 m^2^·g^−1^ protein in the control and US + enzyme treatments) indicate strong emulsifying capacity, making these proteins promising natural emulsifiers for salad dressings, plant-based milks, and sauces. The enhanced gelation behavior, particularly in the US + enzyme and enzyme + US treatments (G′ up to ∼700 Pa), suggests suitability as textural agents in dairy-free yogurts, puddings, and plant-based cheese analogs. These properties, combined with the fiber co-extracted during the process, offer additional nutritional benefits, positioning the extracted proteins as multifunctional ingredients in clean-label, high-fiber, allergen-free food products.

### 3.5 Effect of extraction method on surface color

**Fig. 5:**
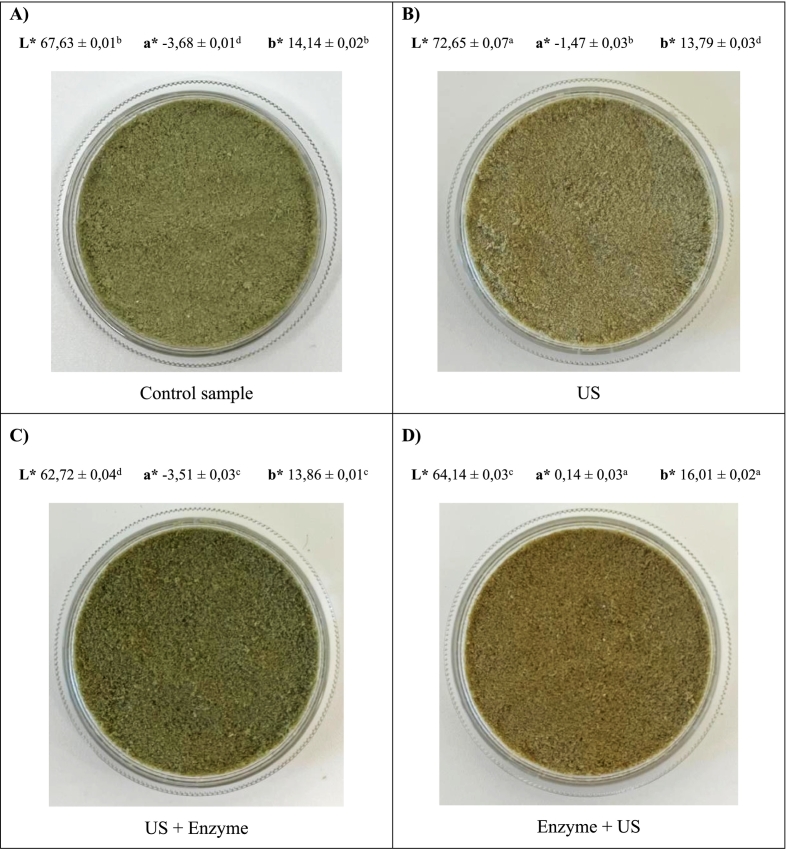
Surface color of the isolated proteins in the presence of Viscozyme®, US or their combinations. The statistical analysis was carried out using one-way ANOVA; US: ultrasound. Different letters in the same color coordinates indicate statistically significant differences (*n* = 3; *p* < 0.05; Tukey test).

Color is an essential sensory index, which is regarded as the initial impression for the consumers. There were significant differences (*p* < 0.05; Fig. 5) among all the protein samples with the terms L*, a*, b*.

US treatment increases lightness significantly, while enzyme treatment darkens the protein samples. Regarding the red-green axis, Enzyme + US treatment shifts the color toward red, while US alone reduces greenness. It can also be seen that enzymatic treatment followed by US increases yellowness (b*: yellow-blue axis), while US alone slightly reduces it. In general, ultrasound treatment makes oat protein lighter and less green, while enzyme treatment (especially before ultrasound) darkens the protein, reduces greenness, and increases yellowness.

Ultrasound has been shown to have negligible effects on color attributes (Zhao et al., 2020). However, other researchers have demonstrated that it increases transparency and reduces turbidity, contributing to a lighter appearance (Li & Xiong, 2021), which is in line with the results observed in this study. The ultrasound waves create micro-channels and cavitation effects, which could have modified the protein properties by enhancing intermolecular hydrogen bonding and reducing crystallinity. This results in a more uniform and regular surface, which can reflect light better, making the extract appear lighter (Wiktor, Sledz, Nowacka, Rybak, & Witrowa-Rajchert, 2016). The protein darkening after extraction with Viscozyme® could be due to breakdown of cellulose and hemicellulose, which releases phenolic compounds and other pigments that contribute to darker and more yellow hues (Chen, Shi, & Hu, 2016).

### 3.6 Effect of extraction method on phytate removal

**Fig. 6:**
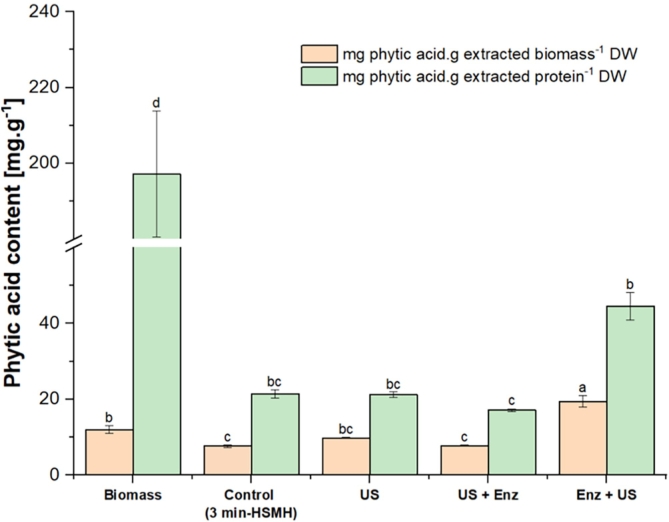
Phytic acid content of isolated proteins in the presence of Viscozyme®, ultrasound (US) or their combinations. Different small letters indicate statistically significant differences (*p* < 0,05; Tukey test).

The effect of pretreatment on the phytate content of extracted oat proteins is shown in Fig. 6. The results indicate that pretreatment significantly affects the phytate content in the biomass and relative to protein content (*p* < 0.05). In first instance, the results show that phytic acid strongly binds to proteins, as evidenced by its increased concentration after protein extraction. In the initial biomass, the phytate content was 11.97 mg.g^−1^ DW, whereas the phyate content relative to the protein content on the same biomass was 197.19 mg.g^−1^ DW. On the extracted biomass, it increased to between 17.11 and 55.56 mg.g^−1^ DW, relative to the protein content. Phytic acid is primarily stored as globoids within protein vacuoles. This interaction is pH-dependent, and the presence of minerals can also influence the protein–phytic acid association (Pragya, Singh, Tiwari, Chouayekh, & Singh, 2025). The phytate content in the initial biomass was significantly lower than the inositol phosphate content reported by Mäkelä, Sontag-Strohm, Olin, and Piironen (2020) for various oat products, which ranged from 27 to 29 mg.g^−1^ DW.

Among all treatments, the combination of US followed by Viscozyme® resulted in the lowest phytate content relative to protein concentration in the extracted biomass (17.12 mg of phytate per g of protein DW). This was followed by the control sample, the sample pretreated with US and with Viscozyme®, which showed an average concentration of 21.60 mg of phytic acid per g of extracted protein. In contrast, the application of Viscozyme® followed by US yielded the highest phytate content per g of protein in the extracted biomass (44.56 mg.g^−1^ protein DW). The phytate content in the protein fraction extracted from the oat fiber pretreated with US and its similar content to the control sample can be explained by the shielding effect of the rigid cell wall, which prevents the cavitation effect from effectively disrupting the protein-phytate complex, hence no significant decrease was evidenced. In the same line, the slightly higher phytate content in the protein fraction extracted with Viscozyme® by alone demonstrates that the degradation of the rigid cell liberates protein-phytate complex to the extraction medium.

This is further corroborated by the lower phytate content in the protein fraction extracted under the synergistic effect of US followed by Viscozyme®. In this case, it could be that the initial ultrasonic disruption likely enhanced enzyme accessibility and facilitated more effective phytate reduction. Given that US can disrupt protein aggregates and alter protein structures, it is plausible that ultrasound could disintegrate phytate, but also disrupt protein-phytate complexes, potentially facilitating the separation of phytate from proteins, as has been demonstrated in canola protein isolates (Karimi, Choi, Samaranayaka, Bhowmik, & Chen, 2025).

Conversely, when enzymatic hydrolysis precedes ultrasound, enzymes may release and solubilize phytate–fiber complexes, but ultrasound can then promote co-precipitation with proteins or strengthen phytate–protein interactions. Additionally, enzymes may break down cell wall barriers, allowing more phytates to enter the protein-rich fraction.

When the phytic acid was analyzed on the biomass extracted, all extraction procedures, except for the enzyme followed by ultrasound (Enzyme + US) treatment, resulted in a reduction in phytate content relative to the starting material. The control extraction, involving alkaline solubilization at pH 11 and isoelectric precipitation without any additional pretreatment, led to a significant reduction in phytic acid content, lowering it to around 8 mg.g^−1^ DW. This can be attributed to the partial solubilization or precipitation of phytate complexes during the extraction process, as phytates tend to dissociate from protein at pH above 9 and form insoluble salts with multivalent cations and proteins at their isoelectric point (Bye, Cowieson, Cowieson, Selle, & Falconer, 2013). The US-only treated sample showed slightly higher phytate content (∼10 mg.g^−1^ DW) than the control, suggesting that while they promoted structural disruption and potential release of proteins, they may have also co-extracted some residual phytates from the oat matrix. Interestingly, the combined treatment of ultrasound followed by enzymatic hydrolysis retained relatively low phytate levels, similar to the control. This suggests that ultrasound effectively disrupted the fiber–phytate matrix and enhanced enzyme accessibility, possibly leading to greater phytate solubilization and/or removal during the downstream precipitation step (Zhang et al., 2021). In contrast, the enzymatic treatment followed by US treatment resulted in a significantly elevated phytic acid content, exceeding 19 mg.g^−1^ DW, higher even than the starting oat fiber material. This unexpected increase suggests a strong dependency on treatment sequence: when enzymatic hydrolysis is applied first, phytate–fiber complexes may be released and solubilized, but subsequent ultrasound may cause co-precipitation of these liberated phytates with proteins or enhance binding between phytates and exposed protein groups. The increased phytate content in the enzyme-followed by-US sample could also be due to enhanced disintegration of cell wall barriers by the enzyme, facilitating phytate release into the protein-rich fraction (Ampofo & Ngadi, 2022). The subsequent ultrasound treatment might induce unfolding of proteins, exposing more positively charged residues (*e.g.*, lysine and arginine) that can strongly bind to the negatively charged phytate molecules (Bye et al., 2013), leading to higher retention in the final isolate. These results highlight the critical role of pretreatment sequence in determining not just protein recovery and functionality, but also the nutritional quality of the extract, particularly in relation to antinutritional compounds like phytates.

Collectively, the findings indicate that US + Enzyme and control treatments are more effective at minimizing phytate co-extraction in oat protein isolates, while Enzyme + US increases phytate retention, likely due to structural and binding changes that enhance phytate–protein interactions. Optimization of processing parameters should therefore consider both protein yield, protein functionality and phytate removal, especially for applications in food where mineral bioavailability is a concern.

The low phytate content achieved in the US + Viscozyme® treatment (17.12 mg·g^−1^ protein) and control extraction (∼21.6 mg·g^−1^ protein) positions these oat protein extracts as particularly suitable for nutritional applications where mineral bioavailability is important, such as fortified plant-based beverages, infant formulas, and high-protein supplements. Reduced antinutritional factors enhance the absorption of essential minerals (*e.g.*, iron, zinc, calcium), making these extracts attractive for clean-label, nutrient-dense food products. In contrast, the higher phytate levels observed in the Enzyme + US treatment (44.56 mg·g^−1^ protein) would require additional processing (*e.g.*, phytase treatment) for similar applications, highlighting the importance of pretreatment sequence in determining both functional and nutritional quality.

## 4 Conclusion

In conclusion, the strategic combination and sequencing of ultrasound and enzymatic pretreatments enable a substantial increase in oat protein yield and functionality from a fiber-rich side stream, transforming an underutilized by-product into a high-value plant-protein ingredient with enhanced emulsifying, gelling, and rheological properties. The higher protein recovery, improved emulsion stability, and stronger gel networks achieved when ultrasound was followed by enzymatic treatment, highlight the synergistic, sequence-dependent disruption of cell-wall barriers and controlled desorption and modification of protein structure. Equally important from a practical perspective is the ability of specific pretreatment sequences to minimize co-extraction of phytic acid, thereby improving potential mineral bioavailability of the final ingredient, whereas other sequences favor superior emulsifying and gelling performance. These findings provide food manufacturers with flexible, scalable tools to tailor oat protein isolates for diverse applications such as meat analogues, dairy alternatives, dressings, or bakery products.

Future work will focus on evaluating the technological performance and sensory impact of these isolates in real food matrices, conducting full-scale pilot trials, and assessing their nutritional quality *in vivo* to accelerate their commercial adoption as sustainable, clean-label protein ingredients.

## CRediT authorship contribution statement

**José Villacís-Chiriboga:** Writing – original draft, Investigation. **Helga Guðný Elíasdóttir:** Writing – original draft, Methodology, Investigation, Formal analysis, Conceptualization. **Isabel Badager:** Writing – review & editing, Methodology, Investigation. **Mehdi Abdollahi:** Writing – review & editing, Writing – original draft, Supervision, Resources, Project administration, Methodology, Funding acquisition, Conceptualization.

## Declaration of competing interest

The authors declare that they have no known competing financial interests or personal relationships that could have appeared to influence the work reported in this paper.

## Data Availability

Data will be made available on request.
